# Social functioning in adults with visual impairment from minority ethnic communities in the United Kingdom

**DOI:** 10.3389/fpubh.2024.1277472

**Published:** 2024-02-01

**Authors:** Nikki Heinze, Lee Jones

**Affiliations:** ^1^BRAVO VICTOR, London, United Kingdom; ^2^UCL, Institute of Ophthalmology, London, United Kingdom

**Keywords:** BAME, inequalities, minority ethnic, sight loss, social functioning, social participation, social relationships, visual impairment

## Abstract

**Background:**

Visual impairment (V.I.) has been associated with a negative impact on social functioning, while social support can impact on well-being in those with V.I. Adults from minority ethnic communities (MEC) are projected to make up an increasing proportion of adults living with V.I. in the UK, but limited research has explored their social functioning. This article provides a preliminary insight into social functioning among MEC adults living with V.I. in the UK.

**Methods:**

The article reports findings from a secondary analysis of V.I. Lives survey data. V.I. Lives was a UK telephone survey, which explored the life experiences of people with V.I. across a wide range of topics including social functioning. This secondary analysis explored social participation, support, isolation, and relationships among a matched control sample of 77 MEC and 77 adults aged 18 and over from White communities (WC). Participants were matched on age, gender, UK region and urban/rural setting. Subgroup analyses were also conducted for the two largest subgroups within the MEC group, Asian (*n* = 46) and Black participants (*n* = 22).

**Results:**

Contact with like-minded people (*U* = 2174.50, *p* = 0.003, *r* = −0.24) and opportunities to take part in more social activities (*U* = 2253.50, *p* = 0.007, *r* = −0.22) was significantly more important to MEC than WC participants. Moreover, MEC participants were significantly less likely to feel supported by friends/family (*U* = 3522.50, *p* = 0.017, *r* = 0.19) and had fewer people they could ask for help (*U* = 3775.50, *p* = 0.001, *r* = 0.26), but there were no significant differences in the perceived impact of V.I. on their friendships/social life and marriage/relationship, their ability to take part in a range of activities, nor their marital status. Asian participants were significantly more likely than Black participants to feel cut off from the people and places around them (*U* = 655.50, *p* = 0.042, *r* = 0.25). Effect sizes were overall small. Although there were no further statistically significant differences between the two groups, Asian participants were also less likely to be able to take part in activities, and more likely to report a negative impact on their social life/friendships and on their marriage/relationship, as well as a smaller social network.

**Conclusion:**

The findings suggest that V.I. may have had a greater impact on social functioning among Asian participants in this sample, including on experiences of social isolation and participation in social activities. Future research will need to confirm these findings and explore the possible reasons.

## 1 Introduction

An estimated 2 million people in the UK have visual impairment (V.I.) and this number is projected to increase to approximately 4 million by 2050 (1). V.I. has been associated with a negative impact on a wide range of life domains, including activities of daily living (2, 3) and participation in sports and leisure activities (4, 5). Moreover, V.I. has been associated with poorer quality of life, mental health outcomes and social functioning (6–11). For instance, findings from the Canadian Community Health Survey Healthy Aging 2008/2009 showed that participation across a range of social activities was lower among older adults (aged 65 and over) with V.I. than those without V.I. (12). Similarly, a review of the psychosocial consequences of diabetic retinopathy, an eye condition which can lead to progressive, irreversible sight loss if left untreated, identified negative impacts on forming and maintaining intimate relationships and family functioning, with higher divorce rates noted among those with diabetic retinopathy compared to the general population (6). In addition, family relationships were found to be impacted by shifting family roles and perceived excessive fussing by family members (6, 8).

Social support, such as instrumental and emotional support from family and friends can have a positive impact on the mental health and well-being of people with V.I. (13–15). Indeed, research from China found that social support from friends mediated the impact of V.I. on depressive symptoms in a probability-based sample of 1,093 older adults (aged 60 and over) (16). In contrast, overprotection, negative support (including unhelpful exchanges, conflict and lack of understanding of the needs and capabilities of individuals with V.I.) or lack of support may have the opposite effect (14, 17). There is conflicting evidence relating to the availability of social support to adults with V.I. Findings from the Canadian Longitudinal Study on Aging (18) showed that, among adults aged 45 to 89, vision loss (having self-reported “fair” or “poor, non-existent, or blind” eyesight with glasses or lenses) was independently associated with lower availability of different types of social support, particularly in those aged 45–64. Social support in this study included overall support, emotional-informational support, positive interactions, as well as affectionate and tangible support. In contrast, research from Netherlands found higher levels of perceived social support in a sample of older adults aged 57 or over with low vision compared to a general population reference group (19). In this study, social support included everyday support (e.g., social companionship and daily emotional support), instrumental, informative and emotional support in problem situations, and support which results in self-esteem and approval. Similarly, research from Jordan found higher levels of perceived social support from family, friends, and significant others among adolescents (aged 12–17) with than without V.I. (20). The authors suggest that this may reflect cultural differences in values and conceptualisations of social support relative to Western cultures, highlighting the need for research which takes into account ethnic backgrounds. Vision loss has also been associated with lower social network diversity among men but not women, reduced social participation and loneliness (18). Indeed, there is evidence of increased loneliness, particularly among older adults with V.I. (18, 21–23), and social isolation. A review of the psychosocial consequences of diabetic retinopathy (6), for instance, found increased social isolation resulting from no longer being able to drive, negative attitudes and stigma, and practical difficulties associated with taking part in social activities.

Adults from minority ethnic communities (MEC) are projected to make up an increasing proportion of adults living with V.I. in the UK (24). Minority ethnic communities include ethnic communities other than the majority group. For instance, in the UK the term tends to encompass people from Asian, Black, mixed and other ethnic but not White backgrounds. Older MEC adults in the UK may be at increased risk of social isolation as a result of health, social and economic inequalities as well as discrimination and racism (25). There is evidence of higher levels of loneliness among older adults from certain ethnic communities in the UK. Victor et al., (26) found that between 24% and 50% of older adults from Chinese, African, Caribbean, Pakistani and Bangladeshi communities reported being always or often lonely compared to 9% of older adults in the UK general population, while levels of loneliness among Indian communities were similar to the general population.

Despite the potential impact of ethnicity on social isolation and loneliness, a recent rapid evidence review of academic and grey literature found limited evidence relating to the social functioning of MEC adults with V.I. (27). Although one review identified unmet needs relating to self-esteem and social isolation among MEC adults living with V.I. (28), the review drew on relatively old data sources and did not elaborate on this. Similarly, qualitative research (29) suggested that older adults with V.I. experienced difficulties with a diminishing social network regardless of ethnicity, but older MEC adults were more likely to have help from family members with everyday tasks than older adults from White communities (WC). The rapid evidence review did not identify research relating to the impact of V.I. on social relationships or wider social participation among minority ethnic communities in the UK. Social connectedness vis-à-vis social relationships and social participation have been associated with a positive impact on physical and mental health (30–32), including mortality (33, 34) and cognitive decline (35). At least among older adults, the relationship between social participation and health has been found to be reciprocal (36). In other words, social participation results in better health outcomes and better health results in more social participation. In contrast, loneliness has been associated with a detrimental impact on mental (37–39) and physical health outcomes, including coronary heart disease and stroke (40), mortality (41, 42) and sleep (43–45). Considering the risk of social isolation and loneliness associated with V.I. and ethnicity, it is important to understand, the social participation and relationships of MEC adults with V.I. The purpose of this article is to provide a preliminary insight into the social functioning (participation and relationships) among MEC adults with V.I. and compare social functioning among MEC groups to a matched control sample of WC adults. It forms part of a series of articles exploring the wider life experiences of MEC adults living with V.I. in the UK.

## 2 Materials and methods

This article uses secondary data collected in the V.I. Lives survey, a UK telephone survey of people with V.I. commissioned by the Royal National Institute of Blind People (RNIB), Thomas Pocklington Trust (TPT) and Guide Dogs for the Blind Association (Guide Dogs) (46), who granted access to the anonymized dataset. Full details relating to the survey methods and sample population have been outlined elsewhere (46, 47). Briefly, data collection took place between 17 December 2019 and 23 March 2020, and between 14 August 2020 and 2 November 2020. All data were collected over the phone by the market research agencies Insight Angels and Acumen Fieldwork. Participants were recruited through the Acumen healthcare database of people who agreed to be contacted for market research, local charities, social media, radio adverts, lists provided by RNIB and Guide Dogs, and partner charities such as Age UK. Non-English speakers and those without V.I. were excluded in an initial call. V.I. was assessed using participants’ self-reported registration status, level of near, distance and peripheral functional vision, and legal driving status. Despite their limitations, self-report measures of V.I. are commonly used in UK general population surveys (48) and in V.I. research (49–55). Comparisons of subjective and objective measures of V.I. concluded that, although self-report measures over-identified V.I. to some extent, considering the cost and resources required to conduct objective measures, such as full ophthalmic examinations, they were an acceptable and valid indicator of V.I. (49, 56).

A total of 769 participants aged 13 and over, including 78 MEC and 667 WC adult participants (aged 18 and over), took part in the research.

### 2.1 Materials

The V.I. Lives survey explored a wide range of topics including priority issues, well-being and mobility. Elements of social functioning explored in the survey included social participation and social relationships.

*Social participation*. As part of a larger list of issues, participants were asked to rate the importance of opportunities to take part in more sport and/or leisure activities. Participants were also asked to indicate their ability to take part in social activities (“*such as meeting friends, going to cafes or bars, dining out, going to concerts, watching sport or other cultural events*”), physical activity and hobbies or interests (“*such as for example being part of a choir or a drama group, going to art or language classes or doing hobbies by yourself*”) as much as they would like. The latter set of questions did not prompt participants to reflect on the extent of their social participation in relation to their V.I.

*Social relationships*. Participants were asked to rate the importance of connecting to like-minded people, and to indicate what effect their sight condition has had on their social life and friendships with others and their marriage or relationship with their partner at the time. Marital status was assessed with a single question asking participants to indicate their marital status. Social isolation and support were explored as part of a wider set of questions on attitudes towards life asking participants to indicate to what extent they agreed or disagreed that they felt supported by family and friends and isolated and cut off from people and places around them. Finally, participants were asked the number of people they felt close to, that is, individuals they could count on if they had a problem.

*Ethnicity.* Participants were asked to indicate how they would describe their ethnic background, from a list of response options including White British, White other, Mixed/multiple ethnic groups, Asian/Asian British (“Asian”), Black/African/Caribbean/Black British (“Black”) and Other ethnic group. Those who selected White British or White other were grouped together in a WC groups, while those selecting Mixed/multiple ethnic groups, Asian/Asian British, Black/African/Caribbean/Black British and Other ethnic group were grouped together in a MEC group.

### 2.2 Data analysis

In order to explore ethnic group differences, a matched control sample of adults aged 18 and over was drawn in R (57) based on age, gender and region. The matched sample consists of 77 participants, respectively, in the MEC and WC subgroups. The MEC group consists of diverse ethnic communities which may have very different experiences and needs relating to social functioning. While the survey was not specifically designed to compare individual subgroups and subsample sizes within the MEC group were low, subgroup analyses therefore consisted of comparing MEC to WC participants and comparing the two largest MEC subgroups, participants from Asian (*n* = 46) and Black communities (*n* = 22). Data analysis was conducted in SPSS (58). For each variable, response distributions were calculated including counts (*n*) and proportions (%). Although questions using Likert-type scales were treated as ordinal, response distributions are reported as counts and proportions rather than medians. Group differences and associations were assessed using Mann–Whitney *U* test for ordinal variables, and chi-square test, or Fisher’s exact test where test assumptions of expected cell counts were violated, for nominal variables. Fisher’s exact tests were conducted using R. Age was the only continuous outcome variable in this article, however, it was not normally distributed as assessed by Shapiro–Wilk test for MEC (*p* < 0.01), WC (*p* < 0.01) and Asian participants (*p* < 0.05) and the non-parametric Mann–Whitney *U* test was used instead of t-tests for both group comparisons. A significance level of *p* = 0.05 was used throughout.

## 3 Results

### 3.1 Participant characteristics

Table 1 provides an overview of the characteristics of the 46 Asian, 22 Black, 77 MEC and 77 WC participants. There were no statistically significant differences between MEC and WC, nor between Asian and Black participants. Mean age was similar for MEC (*M* = 40.78, *SD* = 15.58), WC (*M* = 41.09, *SD* = 15.62), Asian (*M* = 40.17, *SD* = 14.61) and Black participants (*M* = 39.18, *SD* = 14.70). A majority across all groups were female, employed, and residing in a city or big town, specifically in London. While MEC, WC and Asian participants were mainly educated to undergraduate degree level and had severe V.I., a majority of Black participants were educated to Master’s/PhD-level and had moderate V.I.

**Table 1:** Participant characteristics by subgroup.

	Asian (*n* = 46)	Black (*n* = 22)	MEC (*n* = 77)	WC (*n* = 77)
	% (*n*)	% (*n*)	% (*n*)	% (*n*)
Age	*U* = 500.50, *p* = 0.942		*U* = 2919.50, *p* = 0.871	
*M (SD)*	40.17 (14.61)	39.18 (14.70)	40.78 (15.58)	41.09 (15.62)
Range	18–74	18–75	18–85	18–85
Gender	Χ^2^ (1, 68) = 0.49, *p* = 0.482		Χ^2^ (1, 154) = 0.00, *p* = 1.00	
Female	50.0 (23)	59.1 (13)	51.9 (40)	51.9 (40)
Male	50.0 (23)	40.9 (9)	48.1 (37)	48.1 (37)
Region	*p* = 0.789		*p* = 0.344	
London	41.3 (19)	59.1 (13)	44.2 (34)	31.2 (24)
South East	4.3 (2)	9.1 (2)	6.5 (5)	2.6 (2)
South West	6.5 (3)	–	5.2 (4)	3.9 (3)
East of England	6.5 (3)	4.5 (1)	5.2 (4)	2.6 (2)
East Midlands	2.2 (1)	4.5 (1)	3.9 (3)	5.2 (4)
West Midlands	6.5 (3)	–	5.2 (4)	2.6 (2)
North East	–	–	–	5.2 (4)
North West	17.4 (8)	9.1 (2)	13.0 (10)	23.4 (18)
Yorkshire & the Humber	4.3 (2)	4.5 (1)	3.9 (3)	3.9 (3)
Scotland	4.3 (2)	9.1 (2)	7.8 (6)	9.1 (7)
Wales	4.3 (2)	–	3.9 (3)	7.8 (6)
Northern Ireland	2.2 (1)	–	1.3 (1)	2.6 (2)
Setting	*p* = 0.234		Χ^2^ (2, 154) = 4.68, *p* = 0.097	
City/big town	67.4 (31)	77.3 (17)	67.5 (52)	55.8 (43)
Small town	26.1 (12)	9.1 (2)	22.1 (17)	37.7 (29)
Rural area	6.5 (3)	13.6 (3)	10.4 (8)	6.5 (5)
Education^a^	*U* = 518.00, *p* = 0.086		*U* = 2794.00, *p* = 0.397	
No formal qualifications	–	–	–	5.2 (4)
GCSE/O-level	15.2 (7)	4.5 (1)	11.7 (9)	14.3 (11)
A-Level /advanced highers	15.2 (7)	9.1 (2)	15.6 (12)	18.2 (14)
Apprenticeship, vocational, NVQ/HND	17.4 (8)	18.2 (4)	16.9 (13)	11.7 (9)
Undergraduate degree	30.4 (14)	22.7 (5)	27.3 (21)	31.2 (24)
Masters, PhD	15.2 (7)	31.8 (7)	18.2 (14)	16.9 (13)
Non-UK qualifications	4.3 (2)	–	3.9 (3)	–
Other	2.2 (1)	13.6 (3)	6.5 (5)	2.6 (2)
Employment^b^	*p* = 0.771		Χ^2^ (4, 154) = 0.33, *p* = 0.988	
Employed (including part-time)	41.3 (19)	54.5 (12)	42.9 (33)	40.3 (31)
Self-employed	8.7 (4)	4.5 (1)	6.5 (5)	5.2 (4)
Unemployed	19.6 (9)	9.1 (2)	14.3 (11)	14.3 (11)
Retired	6.5 (3)	9.1 (2)	10.4 (8)	11.7 (9)
Other^b^	23.9 (11)	22.7 (5)	26.0 (20)	28.6 (22)
V.I. severity	*U* = 552.50, *p* = 0.516		*U* = 2951.00, *p* = 0.922	
Severe	41.3 (19)	31.8 (7)	39.0 (30)	44.2 (34)
Moderate	34.8 (16)	40.9 (9)	35.1 (27)	23.4 (18)
Mild	23.9 (11)	27.3 (6)	26.0 (20)	31.2 (24)
Could not be classified	-	–	–	1.3 (1)

a Statistical analysis excludes non-UK qualifications and Other.

b Due to expected frequencies of less than 5 in 5 cells (27.8%), the categories looking after family/home, student, long-term sick/disabled and unpaid work (e.g., volunteering, intern, work experiences) were collapsed into the Other category.

MEC, Minority ethnic communities (excluding White minorities); WC, White communities (including White minorities). Results for Fisher’s exact test are shown as *p*-values only.

### 3.2 Social participation

**Figure 1:**
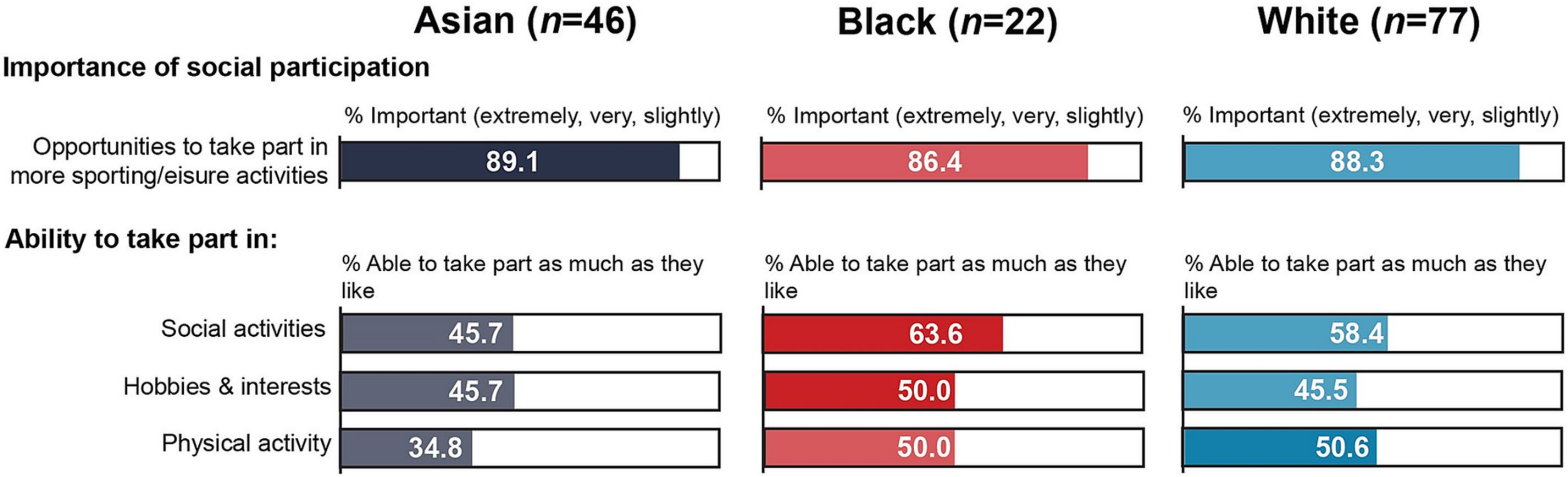
Snapshot of social participation by subgroup.

Table 2 provides an overview of responses relating to social participation and activities. There were no statistically significant differences between participants from Black and Asian communities on any of the social participation variables. Around three quarters in both groups rated the opportunities to participate in more sporting and/or leisure activities as *very* or *extremely important*. Although not statistically significant, participants from Black communities were more likely than those from Asian communities to be able to take part in social activities, hobbies or interests and physical activity (Figure 1). For instance, 63.6% of Black participants were able to take part in social activities as much as they liked compared to 45.7% of Asian participants. In contrast, the proportion of Asian participants who were not able to take part in social activities was almost three times higher than the proportion of Black participants (13.0% vs. 4.5%). Only around one third (34.8%) of Asian participants were able to take part in physical activity as much as they liked, while just over one quarter (26.1%) were not able to take part in physical activity at all.

**Table 2:** Social participation by subgroup.

	Asian (*n* = 46)	Black (*n* = 22)	MEC (*n* = 77)	WC (*n* = 77)
	% (*n*)	% (*n*)	% (*n*)	% (*n*)
Opportunity to participate in more sporting and/or leisure activities	*U* = 509.00, *p* = 0.967		***U* = 2253.50, *p* = 0.007, *r* = −0.22**	
Extremely important	32.6 (15)	31.8 (7)	33.8 (26)	18.2 (14)
Very important	43.5 (20)	45.5 (10)	42.9 (33)	36.4 (28)
Somewhat important	13.0 (6)	9.1 (2)	11.7 (9)	33.8 (26)
Not important at all	10.9 (5)	13.6 (3)	11.7 (9)	11.7 (9)
Ability to take part in…				
Social activities	*p* = 0.541		*p* = 0.361	
Able to take part as much as I’d like	45.7 (21)	63.6 (14)	48.1 (37)	58.4 (45)
Able to take part but not as much as I’d like	37.0 (17)	27.3 (6)	36.4 (28)	33.8 (26)
Not able to take part but would like to	13.0 (6)	4.5 (1)	10.4 (8)	6.5 (5)
Do not want to take part	4.3 (2)	4.5 (1)	5.2 (4)	1.3 (1)
Hobbies or interests	*p* = 0.818		*p* = 0.340	
Able to take part as much as I’d like	45.7 (21)	50.0 (11)	42.9 (33)	45.5 (35)
Able to take part but not as much as I’d like	30.4 (14)	36.4 (8)	35.1 (27)	37.7 (29)
Not able to take part but would like to	17.4 (8)	13.6 (3)	18.2 (14)	9.1 (7)
Do not want to take part	6.5 (3)	–	3.9 (3)	7.8 (6)
Physical exercise	*p* = 0.710		*p* = 0.125	
Able to take part as much as I’d like	34.8 (16)	50.0 (11)	36.4 (28)	50.6 (39)
Able to take part but not as much as I’d like	37.0 (17)	31.8 (7)	36.4 (28)	33.8 (26)
Not able to take part but would like to	26.1 (12)	18.2 (4)	24.7 (19)	11.7 (9)
Do not want to take part	2.2 (1)	–	2.6 (2)	3.9 (3)

MEC, Minority ethnic communities (excluding White minorities); WC, White communities (including White minorities). Statistically significant results are shown in bold. Results of Fisher’s exact test are shown as *p*-values only.

When comparing MEC to WC participants, there was a statistically significant difference in the perceived importance of opportunities to participate in more sporting and/or leisure activities. While the same proportion (11.7%) rated this as *not important at all* in both groups, just over three quarters (76.7%) of MEC participants rated it as being *very* or *extremely important* compared to just over half (54.6%) of WC participants. However, there were no statistically significant associations between ethnicity and the extent to which people were able to take part in social activities, hobbies or interests and physical exercise, although a greater proportion of WC participants reported being able to take part as much as they liked, while MEC participants were more likely to be unable to take part at all in all three types of activities.

### 3.3 Social and romantic relationships

**Figure 2:**
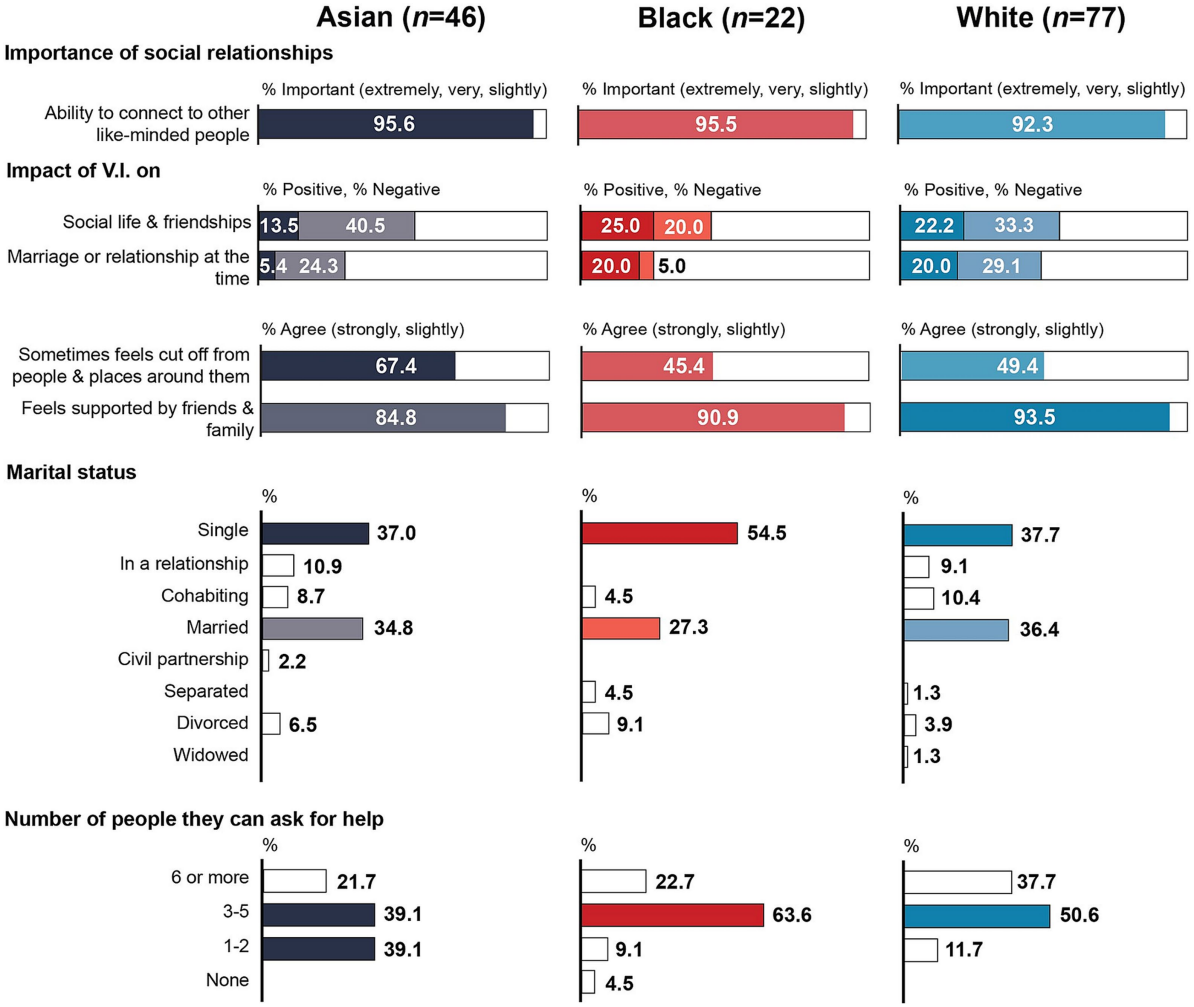
Snapshot of social relationships by subgroup.

Table 3 shows response distributions and statistical comparisons between Asian and Black, and between MEC and WC participants relating to social and romantic relationships. There were again no statistically significant differences between Asian and Black participants relating to the perceived importance of the ability to connect to like-minded people, marital status, the perceived impact of their V.I. on their social life and friendships as well as romantic relationships, feeling supported by friends and family, and the number of people they could ask for help. However, participants from Asian communities were significantly more likely than those from Black communities to feel socially isolated (*p* = 0.042). Just over two thirds (67.4%) of Asian participants agreed that they sometimes felt isolated and cut off from the people and places around them (compared to 45.4% of Black participants), but, likewise, 84.2% of Asian participants agreed that they felt supported by friends and family (Figure 2). Although this was also not statistically significant, V.I. appeared to have had a greater negative impact on participants from Asian communities. This group was twice as likely to report a negative impact on their social life and friendships (40.5% vs. 20.0%) and almost five times more likely to report a negative impact on their marriage or relationship (24.3% vs. 5.0%). In contrast, Black participants were around four times more likely to report a positive impact on their romantic relationships, although a larger proportion of Black participants reported being single (54.5% vs. 37.0%). Only one participant from Black communities reported that they did not have anyone close to them who they could ask for help, Black participants were otherwise more likely than Asian participants to have at least 3–5 people they could draw on for help (86.3% vs. 60.8%), while Asian participants were over four times more likely to have just one or two people to support them (39.1% vs. 9.1%).

**Table 3:** Social relationships by subgroup.

	Asian (*n* = 46)	Black (*n* = 22)	MEC (*n* = 77)	WC (*n* = 77)
	% (*n*)	% (*n*)	% (*n*)	% (*n*)
Ability to connect to like-minded people	*U* = 560.00, *p* = 0.451		***U* = 2174.50, *p* = 0.003, *r* = −0.24**	
Extremely important	41.3 (19)	36.4 (8)	40.3 (31)	20.8 (16)
Very important	39.1 (18)	31.8 (7)	35.1 (27)	33.8 (26)
Somewhat important	15.2 (7)	27.3 (6)	19.5 (15)	37.7 (29)
Not important at all	4.3 (2)	4.5 (1)	5.2 (4)	7.8 (6)
Marital status	*p* = 0.379		*p* = 0.835	
Single	37.0 (17)	54.5 (12)	41.6 (32)	37.7 (29)
In a relationship	10.9 (5)	–	7.8 (6)	9.1 (7)
Cohabiting	8.7 (4)	4.5 (1)	6.5 (5)	10.4 (8)
Married	34.8 (16)	27.3 (6)	31.2 (24)	36.4 (28)
Civil partnership	2.2 (1)	–	2.6 (2)	–
Separated	–	4.5 (1)	1.3 (1)	1.3 (1)
Divorced	6.5 (3)	9.1 (2)	6.5 (5)	3.9 (3)
Widowed	–	–	2.6 (2)	1.3 (1)
Impact of your V.I. on your…				
Social life and friendships with others	Χ^2^ (2, 57) = 2.84, *p* = 0.242		*p* = 0.643	
Positive	13.5 (5)	25.0 (5)	16.7 (11)	22.2 (12)
Negative	40.5 (15)	20.0 (4)	37.9 (25)	33.3 (18)
No effect at all	45.9 (17)	55.0 (11)	45.5 (30)	42.6 (23)
Not relevant	–	–	–	1.9 (1)
Marriage or relationship with your partner at the time	*p* = 0.133		Χ^2^ (3, 121) = 4.40, *p* = 0.221	
Positive	5.4 (2)	20.0 (4)	10.6 (7)	20.0 (11)
Negative	24.3 (9)	5.0 (1)	21.2 (14)	29.1 (16)
No effect at all	59.5 (22)	65.0 (13)	57.6 (38)	40.0 (22)
Not relevant	10.8 (4)	10.0 (2)	10.6 (7)	10.9 (6)
I sometimes feel isolated and cut off from the people and places around me	***U* = 655.50, *p* = 0.042, *r* = 0.25**		*U* = 2557.50, *p* = 0.129	
Strongly agree	37.0 (17)	13.6 (3)	31.2 (24)	22.1 (17)
Slightly agree	30.4 (14)	31.8 (7)	29.9 (23)	27.3 (21)
Neither agree nor disagree	2.2 (1)	9.1 (2)	5.2 (4)	6.5 (5)
Slightly disagree	13.0 (6)	13.6 (3)	13.0 (10)	16.9 (13)
Strongly disagree	17.4 (8)	31.8 (7)	20.8 (16)	27.3 (21)
I feel supported by my friends and family	*U* = 441.00, *p* = 0.339		***U* = 3522.50, *p* = 0.017, *r* = 0.19**	
Strongly agree	52.2 (24)	63.6 (14)	55.8 (43)	74.0 (57)
Slightly agree	32.6 (15)	27.3 (6)	31.2 (24)	19.5 (15)
Neither agree nor disagree	2.2 (1)	–	2.6 (2)	1.3 (1)
Slightly disagree	8.7 (4)	9.1 (2)	7.8 (6)	5.2 (4)
Strongly disagree	4.3 (2)	–	2.6 (2)	–
Number of people you are close to who you could ask for help	*U* = 411.00, *p* = 0.179		***U* = 3775.50, *p* = 0.001, *r* = 0.26**	
6 or more	21.7 (10)	22.7 (5)	20.8 (16)	37.7 (29)
3–5	39.1 (18)	63.6 (14)	48.1 (37)	50.6 (39)
1–2	39.1 (18)	9.1 (2)	28.6 (22)	11.7 (9)
None	–	4.5 (1)	2.6 (2)	–

MEC, Minority ethnic communities (excluding White minorities); WC, White communities (including White minorities). Statistically significant results are shown in bold. Results of Fisher’s exact test are shown as *p*-values only.

When comparing MEC to WC participants, there was a statistically significant difference in the perceived importance of connecting to other like-minded people. Three quarters of MEC participants (75.4%) rated this as *extremely* or *very important*, compared to just over half (54.6%) of WC participants. However, there was no statistically significant association between ethnicity and the perceived impact of V.I. on friendships and social life nor on their marriage or relationship. MEC participants were slightly more likely than WC participants to report a negative impact on their social life and friendships but less likely to report a negative impact on their marriage or relationship. A majority in both groups reported no impact at all on their social and romantic relationships. MEC participants were slightly more likely to be single (41.6% vs. 37.7%) and less likely to be in a relationship, cohabiting, married or in a civil partnership at the time of the survey than WC participants (48.1% vs. 55.8%). Although the proportion of participants who agreed that they sometimes felt isolated and cut off from the people and places around them was higher among MEC participants (61.1% vs. 49.4%), there was no statistically significant difference between the two groups. In contrast, MEC participants were significantly less likely to feel supported by family and friends (*p* = 0.017). Although a majority in both groups agreed that they felt supported by family and friends (87.0% of MEC participants vs. 93.5% of WC participants), MEC participants were twice as likely to disagree on this statement (10.4% vs. 5.2%). There was also a statistically significant difference in the number of people participants were close to and could ask for help: 88.3% of WC participants had at least 3–5 people they could draw on for support compared to 68.9% of MEC participants. Among the latter, 2 reported having no one to draw on for help and over a quarter (28.6%) had 1–2 people.

## 4 Discussion

This article provides a preliminary insight into social functioning in a sample of MEC, including Asian and Black, adults with V.I., comparing social participation and relationships to a sample of age-, gender- and location-matched WC participants.

While there were few statistically significant group differences, social functioning tended to be slightly worse among MEC than WC participants. MEC participants were significantly less likely than WC participants to feel supported by friends and family, have fewer people they could ask for help, and they were more likely to rate better opportunities to take part in social activities and to meet like-minded people as important.

Within the MEC group, participants from Asian communities tended to report worse social participation and relationships than those from Black communities, although there was a statistically significant group difference for perceived social isolation only. Asian participants were significantly more likely to report feeling isolated and cut off from people and places around them than Black participants. But there were no statistically significant differences between the two groups in relation to how supported they felt by family and friends, nor in the number of people they could ask for help, although Black participants tended to have a larger social network. Approximately 8 in 10 Black participants had 3 or more people they felt they could ask for help compared to 6 in 10 Asian participants. In addition, Black participants were slightly more likely to feel supported by friends and family (90.9%) compared to Asian participants (84.8%). As discussed earlier, the quantity and more so the quality of social support can have important implications for mental health and well-being in adults with V.I. (13–15, 17). Indeed, mental well-being was significantly poorer among Asian participants in this sample (59). However, it is not possible to infer causality. As indicated earlier, the relationship between social functioning and health is reciprocal (36). While social isolation may therefore have impacted on mental well-being among Asian participants, it is equally possible that poor mental well-being may have impacted on social functioning in this group. Prior research found that some people from black and minority ethnic communities experiencing mental health difficulties may feel more comfortable accessing informal support provided by friends and family than formal mental health support (60), highlighting the importance of access to these support structures. Awareness and use of more formal support structures provided by eye health services and charities is explored elsewhere in this series (61). The survey did not explore the support quality beyond the extent to which people felt supported by friends and family, nor did it explore the extent to which different types of support, positive and negative, were provided. This should be explored in future research.

While not statistically significant, Asian participants were also more likely than Black participants to report that they were not able to take part in social activities, hobbies and physical activity and to report a negative effect of their V.I. on their social life and relationships and on their marriage or intimate relationships. Though, WC participants were more likely than the other groups to report a negative impact on their intimate relationships and a majority in all groups reported no impact at all on their social and intimate relationships. Previous research has described the impact of sight loss on couples, particularly on shared activities. This includes a need to adapt activities to enable both partners to continue to take part and a shift in responsibilities towards the sighted partner, resulting in perceived dependence and tension in some couples (62). V.I. may also impact on single adults’ ability to form intimate relationships. Research from Israel found that difficulties perceiving non-verbal communication cues and negative attitudes acted as barriers to forming romantic relationships (63). The research suggests that there may be cultural differences in the acceptability of adults with V.I. as a romantic partner, with women with V.I. from traditional Muslim families, for instance, experiencing stigma relating to their suitability from potential romantic partners, the partner’s family and their own families (63). Being in a relationship was associated with living a normal life, increasing self-esteem and reducing loneliness, thus resulting in a positive impact on psychological well-being and self-acceptance (63). However, there is evidence that women with disabilities are more likely to be single or enter relationships at a later age (64). In the current sample, a majority of participants across all groups were single. Although Asian participants were less likely to be single and considerably more likely to be married than Black participants, the proportion of Asian participants who reported being single (37.0%) was higher than the proportion who were never married or in a civil partnership in the general population in England and Wales, where it ranged from 31.7% among Chinese to 20.9% among Pakistani communities (65). Similarly, the proportion of Asian participants who were married (34.8%) was lower than in the general population, where it ranged from 54.4% among Chinese to 63.0% among Pakistani communities (65). In contrast, the proportion of Black participants who reported being married (27.3%) reflects the proportion found in the general population in England and Wales (ranging from 25.5% among Caribbean to 39.8% among African communities) (65). Cultural differences in the importance of marriage and intimate relationships may add additional pressure on Asian participants living with V.I., however, this was not explored in the current survey. Taken together this suggests that social functioning may be more impacted among participants from Asian communities who may be in particular need of support to improve their ability to take part in social activities and strengthen their intimate relationships and social networks. This may be through increasing opportunities to participate in social activities and meet like-minded people.

In contrast, participants from Black communities reported similar or in some cases better social functioning than WC participants. For instance, the former were less likely to report a negative impact of their V.I. on their social life and friendships (20.0% vs. 33.3%), their intimate relationships (5.0% vs. 29.1%), and their ability to take part in social activities (4.5% vs. 6.5%). It is not clear if this reflects greater resilience, better access to support, or self-selection bias whereby people who were doing better and were receiving better support agreed to take part in research. A majority of participants from Black communities were categorised as having moderate V.I. while a majority in all other groups was categorised as having severe V.I. Differences in social functioning may therefore reflect differences in V.I. severity in the individual groups, although evidence for an association between V.I. severity and social participation, for instance, is mixed (66). The survey did not explore the potential reasons for the observed group differences in social functioning, nor the extent to which participants received support to take part in social activities or maintain social relationships. It is possible that cultural differences in attitudes towards those living with V.I. may have impacted on social functioning among the different groups. For instance, as previously mentioned, Muslim women with V.I. in Israel experienced stigma from potential partners, the partner’s as well as their own family relating to their acceptability as a romantic partner (63). In Somalia, V.I. is associated with an inability to do things for oneself and people with V.I. are perceived as not existing (67). Research with Somali refugees with V.I. in the UK found that these perceptions resulted in “learned helplessness” as well as a reluctance to identify as visually impaired and access V.I. support services in the UK among some (67). Although providing care to a family member, particularly a parent, may be considered a family and/or religious duty among some ethnic communities (68), some Somalian refugees expressed reluctance to ask family for support, and/or a preference to ask daughters rather than sons for support (67). Unhelpful perceptions resulting in a reluctance to access formal and/or informal support may ultimately impact on social functioning in this group. As such, attitudes may either directly or indirectly impact on social participation and relationships among adults with V.I. The survey did not explore why participants were unable to take part in social activities nor did it prompt participants to respond in relation to their V.I. It is therefore possible that poor health and comorbidity (rather than V.I.) impacted on social functioning among Asian participants. While Asian participants were almost three times more likely than Black participants to report having bad health, overall comorbidity and presence of comorbid physical health conditions and impairments were higher among Black participants (69). There is some indication that subjective health may be a better indicator of social participation. Research with older adults in Japan found that social participation was impacted by frailty, but social participation was similar for frail older adults with better subjective health as for those who were not frail (70). Poorer perceived health among Asian participants may therefore have had a greater impact on social functioning than the high prevalence of mobility impairments and other conditions among Black participants.

The findings provide a preliminary indication that there may be ethnic group differences in social functioning. More research is required to confirm this in a larger, representative sample and explore reasons for any ethnic group differences, including the roles of attitudes and perceptions, and the impact of subjective versus objective health. Overall, the findings emphasise the need to explore experiences of living with V.I. within individual communities to identify their respective areas of strength and vulnerability and thus enable provision of adequate support.

### 4.1 Limitations

There are several limitations which need to be considered. The findings are based on non-significant differences between small subgroups and cannot be extrapolated to the wider population. The small subsample sizes resulted in a lack of statistical power and future research will need to confirm the findings in larger samples.

This article compares MEC to WC participants and Black to Asian participants. The latter tends to include people from such diverse communities as Chinese, Bangladeshi or Indian, which in itself consists of diverse subgroups such as Hindus, Muslims, Sikhs and Christians (71). By grouping these diverse communities into higher-level subgroups, differences in functioning between subgroups may be lost resulting in a lack of support provided to communities who need it. Future research will need to explore social functioning in more granular ethnic communities to ensure support needs are identified and adequate support can be provided. In addition, the survey excluded non-English speakers who may also be excluded from support where this cannot be provided in other languages. These individuals may be at particular need of support. Future research will need to explore social functioning among adults who do not speak English.

This article focused on group differences based on ethnicity rather than level or onset of V.I. Level of V.I. was assessed using a combination of self-reported registration status or level of near, distance and peripheral functional vision rather than objective measures. Due to the focus of this series of articles on ethnicity and the small subsample sizes in the data set, the impact of differences in level and onset of V.I. within different ethnic groups should be explored in future research. The V.I. Lives survey explored a wide range of topics including elements of social functioning. The current article therefore does not reflect a comprehensive assessment of social functioning including, for instance, the frequency of participation in groups and social activities, or the quality of social support received. Future research will need to explore social participation and associated barriers, as well as social relationships and social isolation in greater depth to gain a more comprehensive understanding of social functioning in ethnic communities.

## 5 Conclusion

The current article explores social functioning in a sample of MEC adults with V.I. While there were few statistically significant group differences, participants from Asian communities tended to report poorer social participation and relationships suggesting that this group may be at greater risk of social isolation than participants from Black and White communities. Although participants from Black communities tended to report similar or in some cases better social functioning than participants from White communities, it is unclear if this reflects greater resilience, better access to support or self-selection bias. Overall, the research points to the importance of exploring experiences in individual ethnic communities rather than higher-level groups to identify areas of unmet need.

## Scope statement

Research has demonstrated the social and health inequalities experienced by minority ethnic communities (MEC), including in relation to eye and vision health. Visual impairment (V.I.) can negatively impact social participation and relationships, whereas appropriate social support can benefit mental health and well-being in individuals with V.I. Yet, there is a lack of evidence relating to the life experiences, including social functioning of MEC adults living with V.I. in the UK. This is important given the diversity of the UK population, wherein MEC adults are projected to make up an increasing proportion of those living with V.I. This article forms part of a series which uses secondary analysis of UK survey data to gain preliminary insights into this under-researched topic. The findings highlight inequalities in social functioning between White and MEC participants, the latter being were less likely to feel supported by friends/family and having fewer people to ask for help. Differences were also observed within the MEC group, with Asian participants being more likely than Black participants to feel socially isolated. Considering the impact of social functioning on well-being, these findings should be of interest to those working in V.I. research and practice.

## Data availability statement

The original contributions presented in the study are included in the article/supplementary material, further inquiries can be directed to the corresponding author.

## Ethics statement

Ethical approval was not required for the study involving humans in accordance with the local legislation and institutional requirements. Written informed consent to participate in this study was not required from the participants or the participants’ legal guardians/next of kin in accordance with the national legislation and the institutional requirements.

## Author contributions

NH: Conceptualization, Data curation, Formal analysis¸ Methodology, Visualization, Writing – original draft. LJ: Validation, Visualization, Writing – review & editing.
